# Clinical Note on a Case of Epithelioma of Tongue in a Young Woman

**Published:** 1906-03

**Authors:** C. Hamilton Whiteford


### CLINICAL NOTE ON A CASE OF EPITHELIOMA OF TONGUE IN A YOUNG WOMAN.

BY

C. HAMILTON WHITEFORD, M.R.C.S., L.R.C.P.

THE patient was a woman, aged 25, married for four years; three children, all healthy, one miscarriage.

*Previous History.*—In August, 1903, the patient noticed that one of the left lower back teeth was rubbing a hole in the side of her tongue, and consulted a medical man, who ordered some medicine and a liniment to be rubbed on the face. In March, 1904, she went to a homœopathic hospital, and had some carious teeth removed, and was given medicine and a paint for the tongue. At the end of September, 1904, she saw another medical man, who considered that she was suffering from what was in all probability epithelioma, and, since the growth was absolutely inoperable, ordered her, on the off-chance of the growth being caused by syphilis, pot. iod. gr. xv. and liq. hydrarg. perchlor. ʒ i. *t.d.s.*, which she took for six weeks. This resulted in only very slight improvement of the tongue condition.

*Present Condition* (November 4th, 1904).—The tongue cannot be protruded in the least, being fixed to the floor of the mouth. Surface of tongue hard, ulcerated in places, especially along left border, with various depressions and hillocks. Beneath the left side of the jaw, extending to within $1\frac{1}{2}$ in. of its angle, is a smooth, firm, apparently solid mass, $1\frac{1}{2}$ in. in horizontal and 1 in. in vertical diameter, fixed to lower margin of jaw and extending under chin across the middle line, to terminate on right side in a hard nodule, $\frac{1}{2}$ in. in diameter, which is attached to the right margin of jaw 2 in. from the middle line.

On November 10th I examined the growth, the patient being under an anæsthetic. A trocar, passed into the under surface of the tongue in the middle line and inserted into the growth in all directions, passed through firm, almost cartilaginous tissue and evacuated no fluid. A needle passed in the same manner gave a similar sensation. The trocar when passed into the mass on the left side of the jaw bored into hard resisting tissue. A piece of growth was snipped off from the tongue for the microscopist, who found that the specimen was composed of squamous cells.

*After History.*—The growth extended, and the patient died on May 8th, 1905.

*Comments.*—The age and sex of the patient made me very loath to admit that the growth was an epithelioma. Exploration of the growth during anæsthesia removed all doubt as to its malignancy, while the microscope demonstrated that the cells of the growth were squamous. Microscopic examination of tumours, while of use for purposes of histological classification, is with our present knowledge (or rather lack of knowledge) of malignant disease totally unable to decide the question of malignancy, of which at present the clinical behaviour of the growth, combined with the naked eye appearance of the fresh section, is the only approximately reliable criterion. Keyser has collected in an interesting paper[1] a large number of cases of epithelioma of the tongue in women. From an average of fourteen groups of statistics, it appears that women are affected to the extent of over 18 per cent. of all cases, which suggests that epithelioma of the tongue is much less rare in women than

[1] *Lancet*, 1904, ii. 829.

is commonly admitted. Personally, in fourteen years of surgical work, I have only seen one other case, and that was in a middle-aged woman, following a gumma of the tongue. Keyser quotes four cases in women under 25 years of age, the youngest being 19. According to Keyser, the chief predisposing causes in women are carious teeth, frequently combined with leucoplacia and syphilis. Smoking does not appear to act as a predisposing cause, since epithelioma of the tongue is not common in women who smoke pipes.

